# Genomic landscape and clinical features of rare subtypes of pancreatic cancer: analysis with the national database of Japan

**DOI:** 10.1007/s00535-023-01986-9

**Published:** 2023-04-07

**Authors:** Tomoki Sakakida, Takeshi Ishikawa, Toshifumi Doi, Ryuichi Morita, Seita Kataoka, Hayato Miyake, Kanji Yamaguchi, Michihisa Moriguchi, Yoshio Sogame, Hiroaki Yasuda, Masahiro Iwasaku, Hideyuki Konishi, Koichi Takayama, Yoshito Itoh

**Affiliations:** 1 Department of Molecular Gastroenterology and Hepatology, Kyoto Prefectural University of Medicine, 465 Kajiicho, Hirokoji Agaru, Kawaramachi Street, Kamigyoku, Kyoto City, Kyoto 602-8566 Japan; 2 Department of Cancer Genome Medical Center, Kyoto Prefectural University of Medicine, Kyoto, Japan; 3 Outpatient Oncology Unit, Kyoto Prefectural University of Medicine, Kyoto, Japan

**Keywords:** Acinar cell carcinoma, Adenosquamous carcinoma, Anaplastic carcinoma of the pancreas, Genome analysis, Pancreatic ductal adenocarcinoma

## Abstract

**Background:**

Special subtypes of pancreatic cancer, such as acinar cell carcinoma (ACC), adenosquamous carcinoma (ASC), and anaplastic carcinoma of the pancreas (ACP), are rare, and so data on them are limited. Using the C-CAT database, we analyzed clinical and genomic characteristics of patients with these and evaluated differences on comparison with pancreatic ductal adenocarcinoma (PDAC) patients.

**Methods:**

We retrospectively reviewed data on 2691 patients with unresectable pancreatic cancer: ACC, ASC, ACP, and PDAC, entered into C-CAT from June 2019 to December 2021. The clinical features, MSI/TMB status, genomic alterations, overall response rate (ORR), disease control rate (DCR), and time to treatment failure (TTF) on receiving FOLFIRINOX (FFX) or GEM + nab-PTX (GnP) therapy as first-line treatment were evaluated.

**Results:**

Numbers of patients with ACC, ASC, ACP, and PDAC were 44 (1.6%), 54 (2.0%), 25 (0.9%), and 2,568 (95.4%), respectively. *KRAS* and *TP53* mutations were prevalent in ASC, ACP, and PDAC (90.7/85.2, 76.0/68.0, and 85.1/69.1%, respectively), while their rates were both significantly lower in ACC (13.6/15.9%, respectively). Conversely, the rate of homologous recombination-related (HRR) genes, including *ATM* and *BRCA1/2,* was significantly higher in ACC (11.4/15.9%) than PDAC (2.5/3.7%). In ASC and ACP, no significant differences in ORR, DCR, or TTF between FFX and GnP were noted, while ACC patients showed a trend toward higher ORR with FFX than GnP (61.5 vs. 23.5%, *p* = 0.06) and significantly more favorable TTF (median 42.3 vs. 21.0 weeks, respectively, *p* = 0.004).

**Conclusions:**

ACC clearly harbors different genomics compared with PDAC, possibly accounting for differences in treatment efficacy.

**Supplementary Information:**

The online version contains supplementary material available at 10.1007/s00535-023-01986-9.

## Introduction

Pancreatic cancer is one of the most lethal malignancies, with a 5-year survival rate of less than 5% [1, 2]. Its incidence has been increasing, and it is now ranked as the world's third leading cause of cancer mortality [3]. Most are cases of pancreatic ductal adenocarcinoma (PDAC), although rare subtypes of pancreatic cancer, such as acinar cell carcinoma (ACC), adenosquamous carcinoma (ASC), and anaplastic carcinoma of the pancreas (ACP, also known as undifferentiated carcinoma), may also be identified. Among all pancreatic cancers, ACC accounts for up to 2% [4–6], ASC for 1–4% [7, 8], and ACP for approximately 1% [9, 10]. While the demographics, clinical characteristics, and prognoses associated with these subtypes are reportedly distinct from those of PDAC, information is limited, with very few reports. According to the results of previous phase III trials, for patients with unresectable PDAC and a good performance status, FOLFIRINOX (FFX) (fluorouracil, folinic acid, irinotecan, and oxaliplatin) or gemcitabine plus nab-paclitaxel (GnP) therapy is recommended as first-line chemotherapy [11, 12]. However, the efficacy of these two regimens remains unclear for the rare subtypes.

As genome analysis using next-generation sequencers has become easier in recent years, it has become evident that the types of genomic mutations detected in cancer tissues differ among cancers, and that these mutations show wide-ranging associations with the development, progression, and treatment of cancer. In Japan, the government established a Center for Cancer Genomics and Advanced Therapeutics (C-CAT) in June 2018, which collects genomic and clinical information on all patients in Japan who have received genomic profiling tests to support hospitals for cancer genomic medicine, and it facilitates the appropriate secondary use of centralized information for future innovative research [13, 14].

In PDAC, activating mutations in the *KRAS* oncogene, and inactivating mutations in tumor suppressor genes such as *TP53*, *SMAD4*, and *CDKN2A*, which are the four major alterations, have been identified [3, 15, 16], and homologous recombination repair (HRR) gene abnormalities such as *BRCA1/2* and *PALB2*, which a subset of them harbors, have attracted attention, accelerating drug discovery for therapeutic targets [17–19]. A recent study reported that 42 resected specimens of Japanese PDAC patients underwent targeted sequencing analyses, and *BRCA1/2* or *PALB2* was detected in 12 (28.6%) cases [20]. Evidence on genomic mutations in rare subtypes of pancreatic cancer from Japan are severely limited, with one report showing *BRCA2* mutations found in 3 of 7 ACC patients [21], though no collective reports on ACP or ASC are available. Therefore, promoting genomic analysis and acquiring insights into the rare subtypes of pancreatic cancer should be further encouraged.

Due to their rarity, there have been no prospective trials involving these special subtypes, and so there is no widespread consensus on treatment. In this study, using the Japanese nationwide C-CAT database, we aimed to analyze the clinical and genomic characteristics of patients with unresectable ACC, ASC, and ACP and compare them with PDAC patients to expand our understanding of these rare subtypes and explore effective chemotherapy.

## Materials and methods

### Study population

We conducted this retrospective observational study using data obtained from the C-CAT database. This national database inclusively aggregates clinical and genomic information on Japanese patients who underwent genomic profiling tests. Namely, basic clinical information such as age, gender, cancer type, pathological diagnosis, and metastatic organs, as well as the chemotherapy regimens given before the genomic profiling test, duration of treatment, best response, occurrence of serious adverse events and information of clinical trials provided by C-CAT. Genomic information includes the genomic variants detected, their variation types, allele frequency and clinical significance, MSI and TMB status. Three genomic profiling tests: OncoGuide™ NCC Oncopanel System (NCC) (Sysmex Co., Ltd., Kobe, Japan), FoundationOne® CDx (F1CDx), and FoundationOne® Liquid CDx (F1L) (Foundation Medicine Inc., Cambridge, USA), have been approved for all solid tumors. Under the National Health Insurance System, patients appropriate for tumor profiling tests include those who have completed or are nearing completion of standard treatment for solid tumors with locally advanced or metastatic disease, and who are considered eligible for chemotherapy after tumor profiling tests. All case data entered from June 2019 to December 2021 for which analysis was available at the time of data update in April 2022 were included. Based on the cancer classification platform OncoTree [22], of all 3,074 cases registered as ‘Pancreas’, 383 cases registered as ‘Cystic Tumor of the Pancreas’ (including Intraductal Papillary Mucinous Neoplasm), ‘Pancreatic Neuroendocrine Carcinoma’, Pancreatic Neuroendocrine Tumor’, ‘Pancreatoblastoma’, ‘Solid pseudopapillary Neoplasm of the Pancreas’, or with unknown histology were excluded. Consequently, the remaining 2,691 cases consisted of those entered as ‘Acinar Cell Carcinoma’, ‘Adenosquamous Carcinoma’, ‘Undifferentiated Carcinoma’, and ‘Pancreatic Adenocarcinoma’ with definite histological diagnosis. This study was approved by the Medical Ethics Review Committee of Kyoto Prefectural University of Medicine (Approval Number: ERB-C-2138) and by the review board of C-CAT (C-CAT Control Number: CDU2021-003N).

### Methods

The following background characteristics and categories related to treatment of the patients were collected using standardized data collection procedures: age, sex, Eastern Cooperative Oncology Group performance status (ECOG-PS), pathological diagnosis, smoking history, history of heavy alcohol consumption, tumor profiling tests, sampling methods and locations, metastatic organs, and chemotherapy regimens with treatment lines. For treatment lines, only regimens for unresectable cancer were counted, and neoadjuvant and postoperative chemotherapies were excluded. To evaluate the treatment efficacy, the overall response rate (ORR), disease control rate (DCR), and time to treatment failure (TTF) associated with each regimen were estimated. ORR was defined as the proportion of all enrolled patients showing a complete or partial response, and DCR was defined as the proportion of all enrolled patients showing a complete response, partial response, or stable disease. These were evaluated based on Response Evaluation Criteria In Solid Tumors version 1.1 (RECIST 1.1) as a guide together with response assessed by the physicians. TTF was defined as the date of the start of treatment to that of treatment discontinuation or death due to any cause. Genomic information was accumulated on representative gene mutations associated with pancreatic cancer, the microsatellite instability (MSI) status, and data on the tumor mutation burden (TMB). To evaluate truly targetable genomic mutations, only variants which were assessed as ‘oncogenic’, ‘pathogenic’, ‘likely oncogenic’, and ‘likely pathogenic’ in the clinical annotation of C-CAT findings were extracted, and variants of unknown significance (VUS) were not included. The clinical annotation of C-CAT was based on the Cancer Knowledge Data Base (CKDB) constructed by C-CAT, which accumulates information on gene mutations, drugs, and clinical trials from public genomic medicine-related databases available worldwide [14]. Due to the nature of the database, which is based on data manually entered by each attending physician, some cases were found to have missing data. Therefore, rates for each variable were based on the number of patients with available data, and analyses were performed including patients with fixed data.

### Statistical analysis

Fisher’s exact test was used to compare all categorical variables and the ANOVA test with Bonferroni correction was used for continuous variables. TTF was estimated with the 95% confidence interval (CI) using the Kaplan–Meier method and compared by the log-rank test. All statistical tests were two-sided and *P* < 0.05 was set as the level of significance. Statistical analyses were conducted using JMP® pro 15 (SAS Institute Inc., Cary, NC, USA) and R version 4.1.2 (R Foundation for Statistical Computing, Vienna, Austria).

## Results

### Frequency of rare-subtype pancreatic cancers and clinical features

**Fig. 1:**
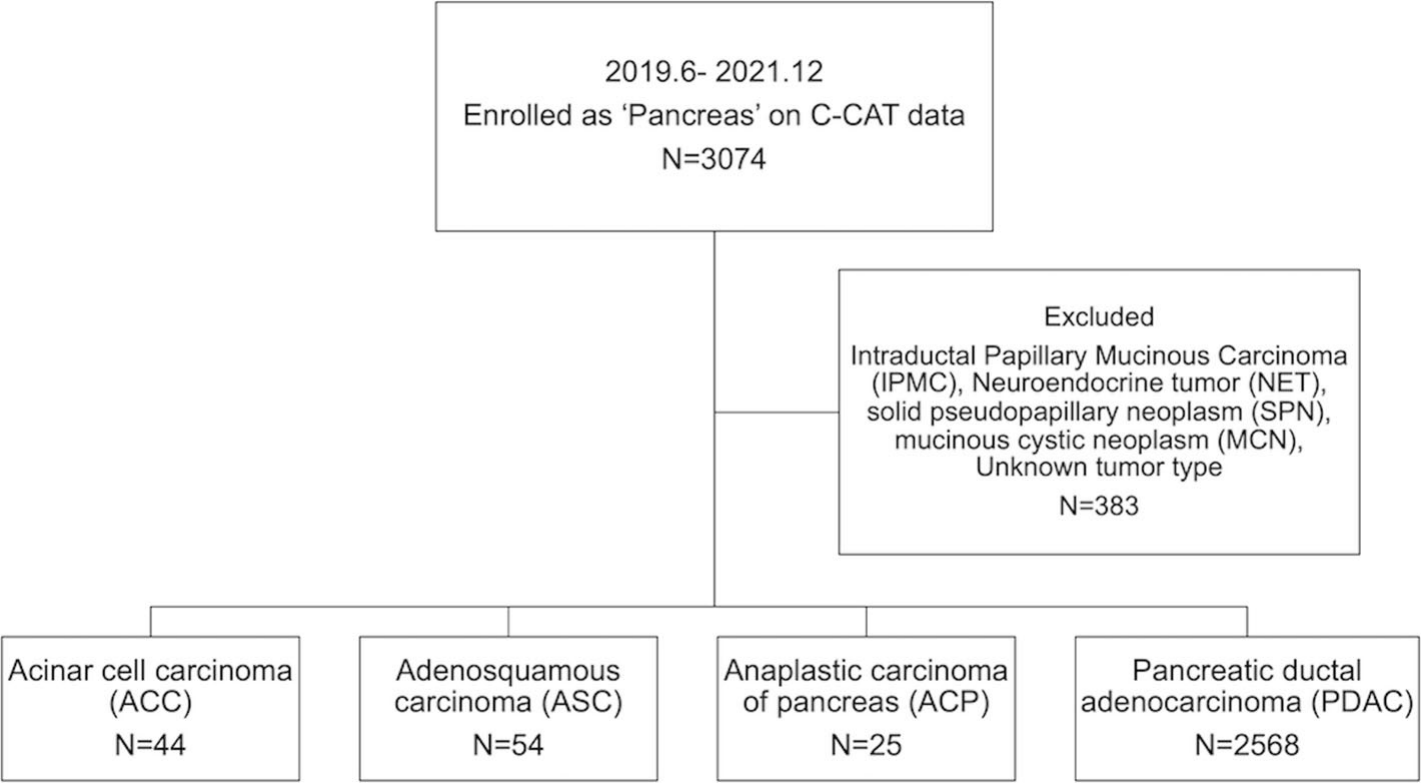
Flow diagram of the study. The diagram displays the tumor types which were excluded and the number of cases of acinar cell carcinoma (ACC), adenosquamous carcinoma (ASC), anaplastic carcinoma of the pancreas (ACP), and pancreatic ductal adenocarcinoma (PDAC), respectively

The group of 2,691 patients in this study was composed of 44 patients (1.6%) with ACC, 54 (2.0%) with ASC, 25 (0.9%) with ACP, and 2,568 (95.4%) with PDAC (Fig. 1). Table 1 summarizes the clinical characteristics of all patients according to their cancer types. On grouping together patients with all four tumor types, the mean age was in the early 60 s, with most having ECOG-PS = 0 or 1. No significant differences were noted among the four groups in terms of age, sex, smoking habit, heavy alcohol consumption, ECOG-PS, sampling method, or site of tumor sampling. Among the genomic profiling tests, F1CDx was the most frequently performed in all groups, followed by NCC, with F1L being the least frequent, with significant differences among the four groups. No notable differences were noted in the proportion of lung and peritoneum metastasis, while the difference in lymph node metastasis was close to significance among the four groups. Moreover, liver metastasis was found in 72.2% of ASC patients, being significantly higher compared with that in PDAC patients (49.5%, *p* < 0.01).

**Table 1:** Clinical characteristics by the subtypes of pancreatic cancer

	ACC *n* = 44	ASC *n* = 54	ACP *n* = 25	PDAC *n* = 2568	*P* value
Age	60.6	64.0	61.5	63.9	0.116
Male *n*, (%)	31 (70.5)	35 (64.8)	11 (44.0)	1455 (56.7)	0.135
Smoking *n*, (%)	21 (47.7)	27 (50.0)	12 (48.0)	1204 (46.9)	0.919
Heavy alcohol consumption *n*, (%)	8 (18.2)	14 (25.9)	3 (12)	340 (13.2)	0.234
ECOG-PS (0/1/2≦) a	26/18/0	34/18/1	16/9/0	1501/913/49	0.990
Genomic profiling test					**0.031**
F1CDx *n*, (%)	34 (77.3)	38 (70.4)	19 (76.0)	1616 (62.9)	
NCC *n*, (%)	7 (15.9)	15 (27.8)	4 (16.0)	585 (22.8)	
F1L *n*, (%)	3 (6.8)	1 (1.8)	2 (8.0)	367 (14.3)	
Sampling method (surgery/biopsy) b	17/24	23/29	15/8	1131/1048	0.378
Sampling area (primary tumor/metastatic site) c	21/20	39/14	17/6	1548/652	0.115
Metastatic organs					
Liver *n*, (%)	26 (59.1)	39 (72.2)	13 (52.0)	1271 (49.5)	0.005 *
Lung *n*, (%)	8 (18.2)	8 (14.8)	3 (12.0)	536 (20.9)	0.562
Lymph node *n*, (%)	17 (38.6)	16 (29.6)	6 (24.0)	576 (22.4)	0.051
Peritoneum *n*, (%)	11 (25.0)	9 (16.7)	9 (36.0)	580 (22.6)	0.282

*ACC* acinar cell carcinoma, *ASC* adenosquamous cell carcinoma, *ACP* anaplastic carcinoma of pancreas, *PDAC* pancreatic ductal adenocarcinoma, *ECOG-PS* Eastern Cooperative Oncology Group Performance Status, *F1CDx* FoundationOne® CDx, *NCC* OncoGuideTM NCC Oncopanel System, *F1L* FoundationOne® Liquid CDx

^a^Data available for 53 patients in ASC, and 2463 patients in PDAC

^b^Data available for 41 patents in ACC, 52 patients in ASC, 23 patients in ACP and 2179 patients in PDAC

^c^Data available for 41 patents in ACC, 53 patients in ASC, 23 patients in ACP and 2200 patients in PDAC

*p < 0.01 between ASC and PDAC with Bonferroni correction. P-values less than 0.05 are shown in bold

### Genomic characteristics

**Fig. 2:**
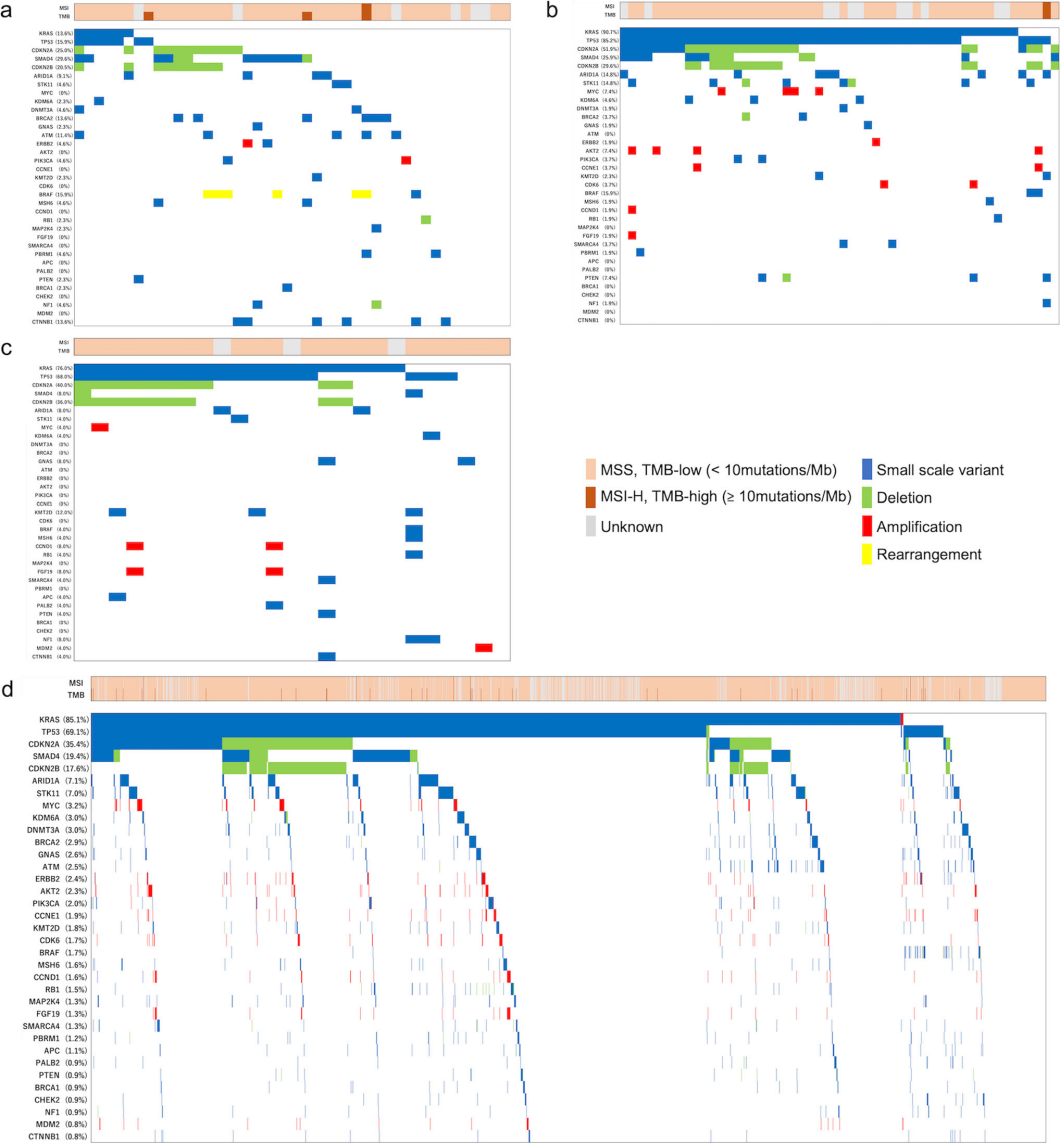
Overview of the most common genomic alterations. Data are shown for **a** acinar cell carcinoma (ACC), **b** adenosquamous carcinoma (ASC), **c** anaplastic carcinoma of the pancreas (ACP), and **d** pancreatic ductal adenocarcinoma (PDAC). The genes most frequently found in PDAC are listed from the top to bottom

**Fig. 3:**
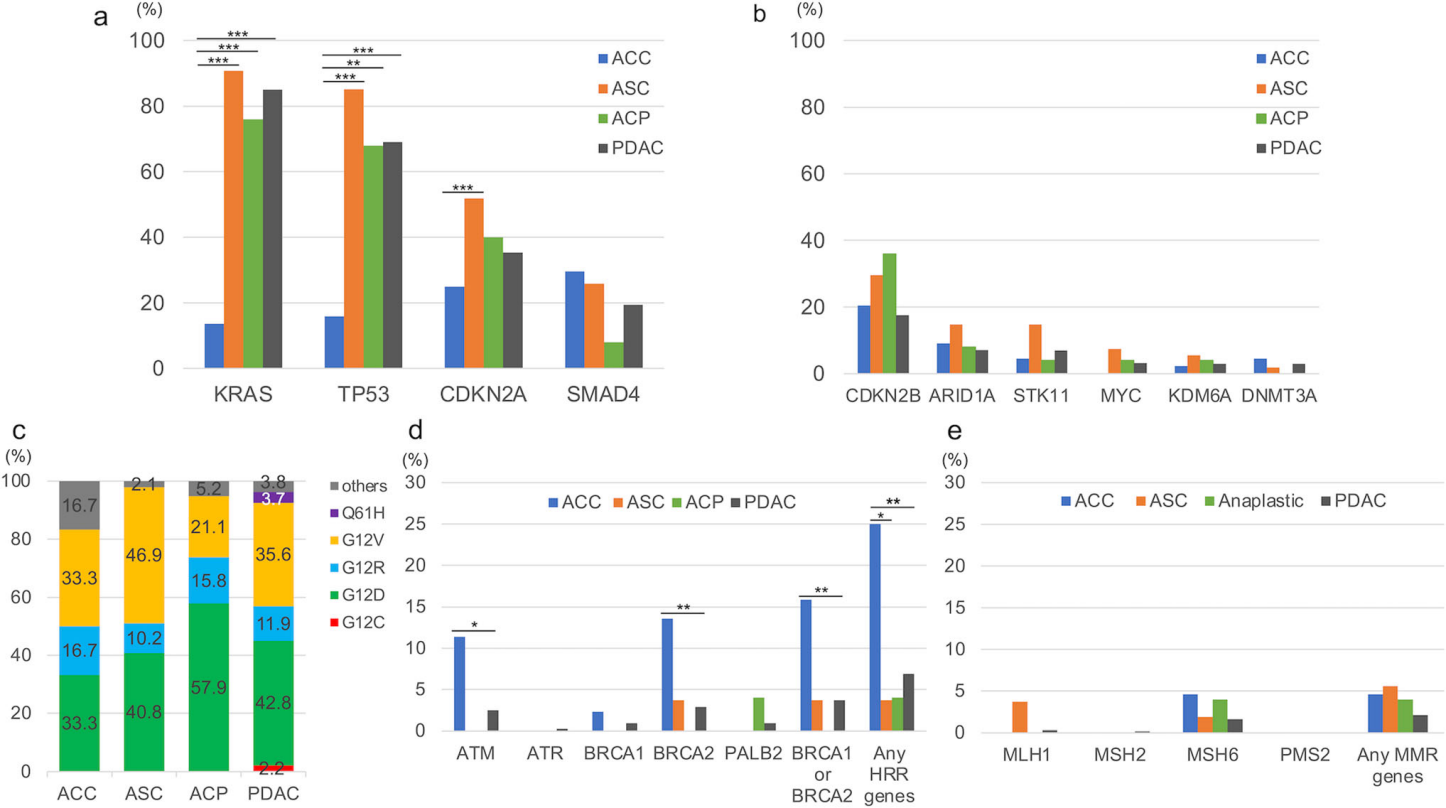
The frequency of major genomic alterations among rare subtypes of pancreatic cancer and pancreatic ductal adenocarcinoma (PDAC). Data are shown for **a** top 4 and **b** 5th-10th gene mutations found in PDAC. **c** shows the distribution of KRAS subtypes, **d** and **e** show the frequencies of the representative homologous recombination repair (HRR) and mismatch repair (MMR) genes among the four tumor types, respectively

Overall genomic alterations (small-scale variant, deletion, amplification, and rearrangement) with the MSI and TMB status of the four tumor types are shown in Fig. 2a–d, and the top 10 genes in PDAC are compared among the four groups by charts shown in Fig. 3a, b. The list of genes following these frequent gene mutations are presented in Supplemental Table 1. In PDAC patients, the most frequently observed variants were *KRAS* (85.1%), *TP53* (69.1%), *CDKN2A* (35.4%), and *SMAD4* (19.4%). ASC and ACP patients showed similar results, with 90.7 and 76.0% for *KRAS*, 85.2 and 68.0% for *TP53*, 51.9 and 40.0% for *CDKN2A*, and 25.9 and 8.0% for *SMAD4*, respectively. In contrast, in patients with ACC, *KRAS* and *TP53* were significantly less frequently detected in comparison with the other three tumor types, at 13.6 and 15.9%, respectively (*p* < 0.01 for *TP53* between ACC and ACP, *p* < 0.001 for the rest). In addition, *CDKN2A* alteration was found in 25.0% of ACC patients, which was also significantly lower than in ASC (*p* < 0.001). For more detail regarding *KRAS*, G12D and G12V were found to be the most common in either group, followed by G12R. G12C, which is well-known for its high detection rate in lung cancer and the recent development of its inhibitor sotorasib [23], was observed only in 2.2% of *KRAS* mutant PDAC patients, and none in ACC, ASC, and ACP patients (Fig. 3c). The genomic variants which followed the above top 4 variants in PDAC patients were *CDKN2B* (17.6%), *ARID1A* (7.1%), *STK11* (7.0%), *MYC* (3.2%), *KDM6A* (3.0%), and *DNMT3A* (3.0%). The proportions of these genomic mutations in ACC, ASC, and ACP patients were as follows: *CDKN2B* (20.5, 29.6, and 36.0%, respectively), *ARID1A* (9.1, 14.8, and 8.0%, respectively), *STK11* (4.6, 14,8, and 4.0%, respectively), *MYC* (0, 7.4, and 4.0%, respectively), *KDM6A* (2.3, 5.6, and 4.0%, respectively), and *DNMT3A* (4.6, 1.9, and 0%, respectively). No differences were observed for these genomic variants.

Representative HRR gene mutations are shown in Fig. 3d. These genes were chosen based on previous reports [24, 25]. In PDAC patients, the frequencies of *ATM*, *ATR*, *BRCA1*, *BRCA2,* and *PALB2* were 2.5, 0.2, 0.9, 2.9, and 0.9%, respectively. HRR genes were generally less common in ASC and ACP patients, with only 3.7% for *BRCA2* in ASC and 4% for *PALB2* in ACP. On the other hand, in ACC patients, the rates for *ATR* and *BRCA1* were not high (0 and 2.3%, respectively), while *ATM* and *BRCA2* were 11.4 and 13.6%, respectively, which were both significantly higher compared with PDAC patients (*p* < 0.05 and *p* < 0.01, respectively). Thus, 15.9% of ACC patients had *BRCA1* or *BRCA2*, and 25.0% had at least one of these five genes, both of which were markedly more frequent in comparison with PDAC patients. Detailed data on the other 13 HRR genes including *BAP1*, *BARD1*, *BRIP1*, *CHEK1/2*, *RAD51B/C/D*, *FANCA/C/L*, *MRE,* and *NBN*, are shown in Supplemental Table 1 (with yellow markers). Considering all these 18 HRR genes, 9.4% of PDAC patients, 25% of ACC patients, 9.3% of ASC patients, and 4.0% of ACP patients had at least one of the HRR genes, showing markedly higher rates in ACC compared with PDAC (*p* = 0.01).

Data regarding mismatch repair (MMR) gene alterations are presented in Fig. 3e. In PDAC patients, the frequencies of *MLH1*, *MSH2*, *MSH6,* and *PMS2* were 0.3, 0.2, 1.6, and 0.04%, respectively. *MSH6* was found in 4.6% of ACC patients, 1.9% of ASC patients, and 4.0% of ACP patients. *MLH1* was detected in 3.7% of patients with ASC. There were no cases of patients with ACC, ASC, or ACP with *MSH2* or *PMS2*.

Other notable gene mutations besides those mentioned above included *BRAF* and *CTNNB1*. *BRAF* alterations was detected in 15.9% of ACC patients, being significantly higher than that of PDAC (1.7%, *p* < 0.001). In particular, *BRAF* fusions were detected in 13.6% of ACC, 0.2% of PDAC, and none of ASC or ACP, indicating that this fusion was present almost exclusively in ACC patients. *CTNNB1* was also a frequently detectable gene in ACC (13.6%), compared with PDAC (0.8%), ASC (0%), and ACP (4.0%). *PTEN* was a variant identified in 7.4% of ASC patients, being significantly more common than in PDAC patients (0.9%, *p* < 0.05). Furthermore, *KMT2D* was found in 12.0% of ACP patients, showing a trend toward a higher prevalence than in PDAC (1.8%) (Supplemental Table 1).

**Fig. 4:**
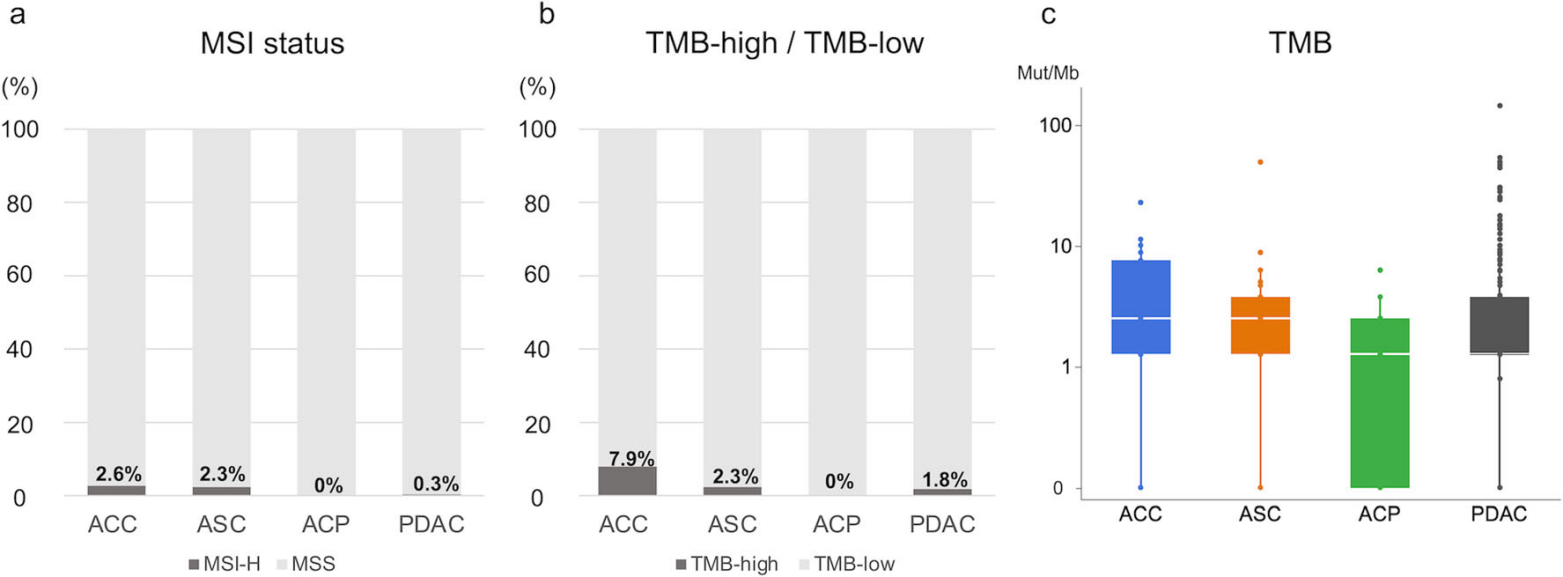
MSI and TMB status among rare subtypes of pancreatic cancer and pancreatic ductal adenocarcinoma (PDAC). The distribution of **a** MSI status, **b** TMB status and **c** TMB load among the four tumor types are shown

Figure 4 shows data on the MSI and TMB status among the four tumor types. These are biomarkers predicting the efficacy of immunotherapy [26, 27]. The proportion of MSI-H was 0.3% in PDAC, compared with 2.6% in ACC, 2.3% in ASC, and 0% in ACP, with no significant differences among the four groups (Fig. 4a). TMB-high (> 10 mutations/Mb) tumors were observed in 1.8% of PDAC, 7.9% of ACC, 2.3% of ASC, and 0% of ACP, with a slightly higher trend in ACC compared with PDAC (*p* = 0.18) (Fig. 4b). Additionally, the median TMB was 2.51 mutations/Mb for PDAC, 3.99 for ACC, 3.93 for ASC, and 1.78 for ACP, showing no significant difference among the four groups (Fig. 4c).

### Treatment response

Table 2 summarizes ORR and DCR of first-line FFX and GnP therapy for each tumor type. Among 1977 PDAC patients, ORRs of FFX and GnP were comparable, at 24.1 and 25.2%, respectively. DCR was 60.3% for FFX and 66.8% for GnP, being significantly higher for the latter (*p* = 0.006). By contrast, in ACC patients, FFX tended to lead to better ORR compared with GnP, approaching significance (61.5 vs. 23.5%, respectively, *p* = 0.06). In addition, DCR for FFX was elevated at 76.9%. ASC and ACP showed no noticeable differences in ORR and DCR between these two regimens.

**Table 2:** Overall response rate (ORR) and disease control rate (DCR) by the subtypes of pancreatic cancer

	FFX	GnP	*P* value
ACC *n* = 28	13	15	
CR *n*, (%)	1 (7.7)	0	
PR *n*, (%)	7 (53.8)	4 (23.5)	
SD *n*, (%)	2 (15.4)	6 (35.3)	
PD *n*, (%)	2 (15.4)	5 (29.4)	
NE *n*, (%)	1 (7.7)	2 (11.8)	
ORR *n*, (%)	8 (61.5)	4 (23.5)	0.06
DCR *n*, (%)	10 (76.9)	10 (58.8)	0.44
ASC *n* = 40	19	21	
CR *n*, (%)	0	0	
PR *n*, (%)	2 (10.5)	3 (14.3)	
SD *n*, (%)	7 (36.8)	10 (47.6)	
PD *n*, (%)	8 (42.1)	7 (33.3)	
NE *n*, (%)	2 (10.5)	1 (4.8)	
ORR *n*, (%)	2 (10.5)	3 (14.3)	1.00
DCR *n*, (%)	9 (47.4)	13 (61.9)	0.53
ACP *n* = 20	5	15	
CR *n*, (%)	0	0	1
PR *n*, (%)	1 (20.0)	4 (26.7)	NA
SD *n*, (%)	2 (40.0)	3 (20.0)	
PD *n*, (%)	0	4 (26.7)	
NE *n*, (%)	2 (40.0)	4 (26.7)	
ORR *n*, (%)	1 (20.0)	4 (26.7)	1.00
DCR *n*, (%)	3 (60.0)	7 (46.7)	1.00
PDAC *n* = 1977	607	1370	
CR *n*, (%)	0	6 (0.4)	
PR *n*, (%)	146 (24.1)	340 (24.8)	
SD *n*, (%)	220 (36.2)	570 (41.6)	
PD *n*, (%)	160 (26.4)	296 (21.6)	
NE *n*, (%)	81 (13.3)	158 (11.5)	
ORR *n*, (%)	146 (24.1)	346 (25.2)	0.61
DCR *n*, (%)	366 (60.3)	916 (66.8)	**0.006**

*FFX* FOLFIRINOX, *GnP* Gemcitabine + nab-pactaxel, *ACC* acinar cell carcinoma, *ASC* adenosquamous cell carcinoma, *ACP* anaplastic carcinoma of pancreas, *PDAC* pancreatic ductal adenocarcinoma, *CR* complete response, *PR* partial response, *SD* stable disease, *PD* progressive disease, *NE* not evaluable, *ORR* overall response rate, *DCR* disease control rate, P-values less than 0.05 are shown in bold

### Time to treatment failure

**Fig. 5:**
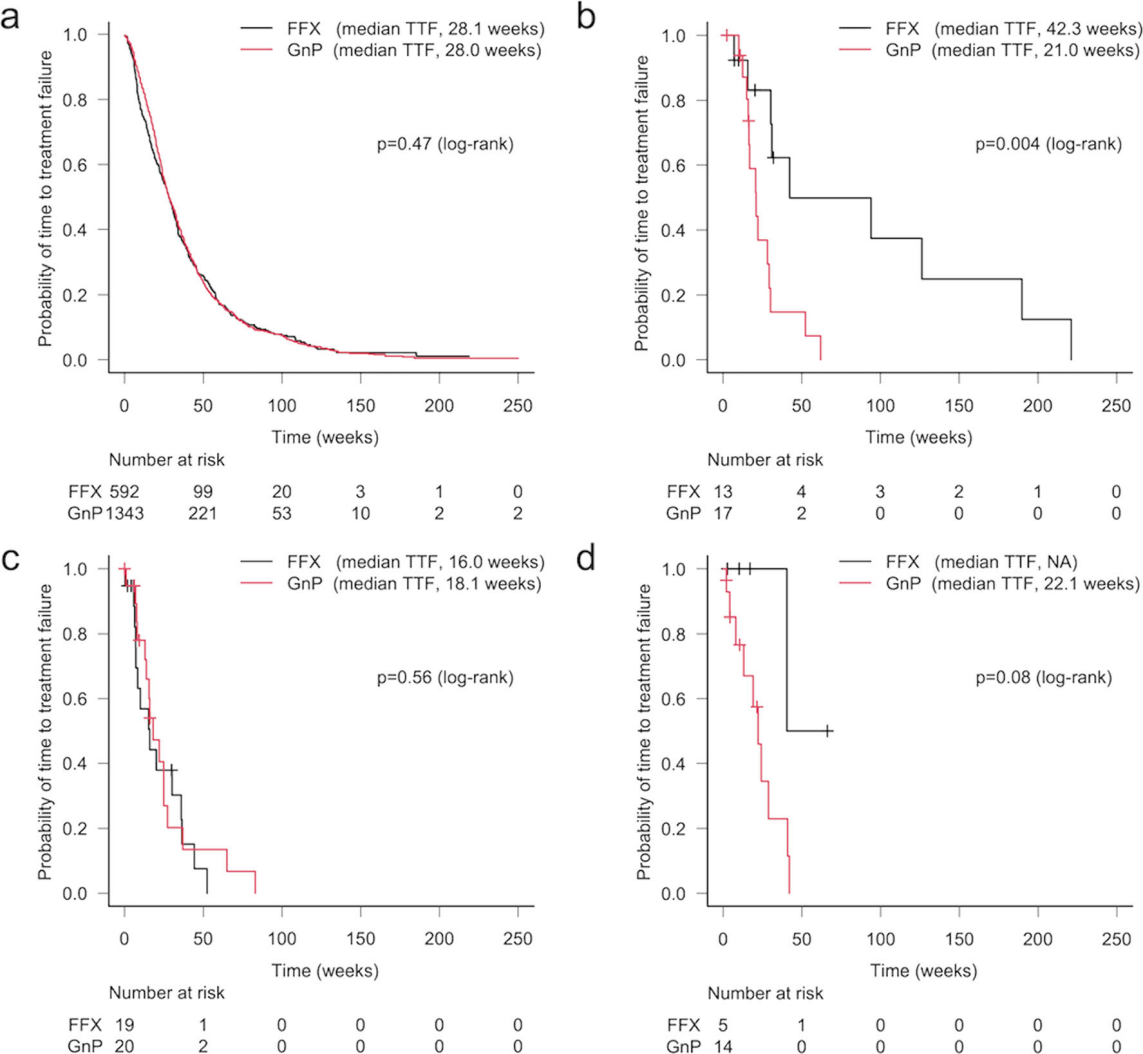
Kaplan–Meier curves of time to treatment failure (TTF) according to first-line FOLFIRINOX (FFX) versus gemcitabine plus nab-paclitaxel (GnP) therapy. Data are shown for **a** pancreatic ductal adenocarcinoma (PDAC), **b** acinar cell carcinoma (ACC), **c** adenosquamous carcinoma (ASC), and **d** anaplastic carcinoma of pancreas (ACP) patients

TTF of first-line FFX and GnP in each tumor group is shown in Fig. 5. In PDAC patients, the median TTF for FFX was 28.1 weeks (95%CI 25.0–30.9 weeks) vs. 28.0 weeks (95%CI 26.7–30.7 weeks) for GnP, resulting in very similar Kaplan–Meier curves (Fig. 5a). On the other hand, for patients with ACC, TTF was longer for FFX (median TTF, 42.3 weeks; 95%CI 15.7–189.9 weeks), whereas the median TTF for GnP was 21.0 weeks (95%CI 16.0–29.3 weeks, *p* = 0.004) (Fig. 5b). ASC patients showed a shorter median TTF on receiving both regimens without any differences; 16.0 weeks (95%CI 7.0–36.0 weeks) for FFX, and 18.1 weeks (95%CI 13.0–24.9 weeks) for GnP. In ACP patients, the median TTF for FFX was not reached (95%CI 40.4 weeks to not reached) and 22.1 weeks (95%CI 8.0–40.9 weeks) for GnP (Fig. 5c, d).

Patients with HRR genes have been reported to show a more favorable response to platinum-containing regimens [28, 29]. Thus, we examined TTF of FFX and GnP among ACC patients according to the presence or absence of HRR genes (regarding the five representative genes mentioned in Fig. 3d). Among ACC patients with HRR genes, FFX showed significantly longer TTF compared with GnP (126.3 vs. 25.7 weeks, respectively; *p* = 0.04, Supplemental Fig. 1a). However, there was also a trend toward longer TTF of FFX compared with GnP in patients without HRR genes, being close to significance (42.3 vs. 20.7 weeks, respectively; *p* = 0.05, Supplemental Fig. 1b). Furthermore, we evaluated the treatment response by prevalence of *BRAF* fusion or *CTNNB1* mutation, characteristic variants of ACC. Patients with *BRAF* fusion genes showed a significantly shorter duration of successful chemotherapy compared with those without them (15.7 vs. 30.1 weeks, respectively; *p* = 0.04, Supplemental Fig. 2a). There was no notable difference in TTF of chemotherapy depending on the presence or absence of *CTNNB1* mutation (36.9 vs. 28.1 weeks, respectively; *p* = 0.74, Supplemental Fig. 2b).

## Discussion

Herein, using the Japanese Nationwide Comprehensive Genomic Profiling (CGP) database, we demonstrated the clinical and genomic characteristics of rare subtypes of pancreatic cancer on comparison with PDAC. Among the three rare subtypes, ASC and ACP exhibited relatively similar genomics to PDAC, whereas ACC harbored a clearly different genomic profile. Namely, *KRAS*, *TP53*, and *CDKN2A*, the most typical abnormalities in pancreatic cancer, were present at a low frequency, and other potentially targetable gene alterations, such as HRR genes and *BRAF* fusion, were more prevalent. This molecular difference may have contributed to the gap in treatment sensitivity between FFX and GnP therapy. Because of their rarity, there are no prospective trials for rare subtypes of pancreatic cancer, and possible effective regimens have only been described in a limited number of retrospective studies and case reports. There are also very few studies that have comprehensively evaluated their genomic profiles. To the best of our knowledge, this is the first report to provide data on the genomic features and clinical benefits of chemotherapy in rare subtypes of pancreatic cancer concurrently, while also exploring the differences from PDAC, based on a large real-world cohort. Another advantage of our study is that we analyzed genomic alterations that were interpreted based on public databases.

Recent studies revealed that ACC is a morphologically and immunohistochemically distinct tumor from PDAC. The low detectability of *KRAS*, *TP53*, and *CDKN2A* was consistent with previous reports, and interestingly, *KRAS* wild patients were enriched with several non-overlapping actionable genomic alterations [30–32]. Comparing these reports with our data, no clear differences in the frequency of genomic alterations among races were found. The high prevalence of *ATM* and *BRCA2*, HRR genes known to be markedly sensitive to platinum-containing regimens and PARP inhibitors [28, 29, 33], may explain the better treatment response to FFX compared with GnP in our study. However, contrary to our expectations, better outcomes with FFX were also noted in patients without HRR genes. Takahashi et al. reported that S-1 monotherapy was more effective than Gemcitabine therapy for ACC, indicating that ACC may be highly susceptible to fluoropyrimidine agents [34]. Moreover, consistent with previous reports, *BRAF* fusions, especially *SND-BRAF* fusion which activates the MAPK pathway, was highly prevalent and a distinctive alteration in ACC [31]. Based on our review of a small number of cases, these patients showed a poor response to chemotherapy. The MEK inhibitor trametinib may be a therapeutic candidate in this population, since activation of the MAPK pathway was abrogated by MEK inhibition in in vitro experiments. Alterations of *CTNNB1*, a WNT-β catenin pathway-related gene, were found in a subset of ACC [30, 35]. Although drugs targeting this pathway are still under development, they may become valuable in the future. Liu et al. reported that up to 14% of ACC harbored MSI/defective MMR, which was higher than in PDAC [36, 37]. In our study, MSI-H and TMB-high tumors were marginally more common than PDAC, raising expectations for immunotherapy in this setting, but further accumulation of evidence is warranted.

ASC has been reported to show a greater metastatic potential and be associated with a poorer prognosis compared with PDAC [38–40]. In the present study, consistent with these reports, the significantly higher number of liver metastases compared with PDAC was a peculiar finding in this subtype. Genomic involvement has been investigated in several studies to elucidate the clinical behavior of ASC. A recent study conducting genome analysis of clinical samples revealed that ASCs are likely to share the most common genomic mutations including *KRAS*, *TP53*, *CDKN2A,* and *SMAD4*, with PDACs, which supports the theory that these tumor types are derived from a common lineage [40]. Our data were in line with these reports, and as a side note, the breakdown of *KRAS* subtypes was also similar to PDAC, with the majority being G12D and G12V followed by G12R. A novel finding was that *PTEN*, one of the key suppressors of the PI3K/AKT/mTOR pathway, was significantly more altered than PDAC. While the molecular impact of *PTEN* abnormalities in ASC remains unclear, PI3K inhibitors may play a role in the anti-tumor effect in the future. Nonetheless, as a harsh reality, GnP and FFX therapy for ASC demonstrated comparable but also limited treatment efficacy in this study, which reinforces the results of previous studies [41, 42].

Even less evidence for ACP is available, based on sporadic case reports and a small number of case series. Histologically, ACP is composed of malignant epithelial and mesenchymal elements that do not exhibit specified differentiation [43]. Previous studies reported that, although morphologically distinct, ACP closely resembles PDAC at the molecular level. The possible mechanism of carcinogenesis is as follows: *KRAS*, *TP53*, *CDKN2A*, and *SMAD4* mutations cause pancreatic intraepithelial neoplasia (PanIN), a precancerous lesion of PDAC, and epithelial-mesenchymal transition (EMT) occurs during the process of invasive growth of tumor cells, whereby pleiomorphic cells are formed, resulting in the development of ACP [43–46]. Indeed, our results were in agreement with this, showing a parallel pattern of genomic alterations with PDAC. Of note, there were no MSI-H/TMB-high cases in our ACP cohort. Since no previous reports on the MSI/TMB status exist in this tumor category, further investigation is required. Regarding studies on chemotherapy, Imaoka et al. demonstrated that paclitaxel-containing regimens such as GnP facilitate favorable outcomes in ACP patients [47]. From our results, disease control was also achieved by FFX therapy; however, due to the small number of cases, it is difficult to conclude which is preferable.

The limitations of our study are mainly attributed to the content of the nationwide C-CAT database and the nature of real-world data. First, the cohort was composed of relatively young patients with good PS, since patients who underwent CGP testing were included, which may have resulted in a selection bias. Second, although the study was based on a nationwide database, due to the rarity of the diseases, the numbers of patients with the rare subtypes were low. Third, due to incomplete data entries, the overall survival, the standard measure of the treatment response, was not available and TTF was used instead. Nonetheless, it should be stated that TTF has become increasingly accepted as a practical endpoint for real-world data and is used in various studies [48, 49]. Forth, we could not evaluate somatic and germline mutations separately, since most analyses were performed by F1CDx, which does not distinguish between them. Lastly, the details of treatment, such as drug withdrawal or dose reduction, were not available. However, we believe that our study is valuable in that we performed genomic analysis using common assays across tumor groups, and discussed it along with their clinical characteristics, within the same time period. Moreover, the comprehensiveness of the C-CAT database, which provides information on almost all patients who underwent CGP in Japan, is also a strength of this study.

In conclusion, each tumor category exhibited different clinical and genomic characteristics compared with PDAC, especially ACC, which showed a completely distinct genomic profile and response to chemotherapy. While rare subtypes of pancreatic cancer are usually excluded from clinical trials, the unique genomic alterations found in this study may encourage participation in basket trials. With the growing dissemination of precision medicine, we hope that our results provide some insights into how to treat rare cancers, which remains challenging.

## Supplementary Information

Below is the link to the electronic supplementary material.

Supplementary file1 (PDF 269 KB)

## Abbreviations

PDAC: Pancreatic ductal adenocarcinoma
ACC: Acinar cell carcinoma
ASC: Adenosquamous carcinoma
ACP: Anaplastic carcinoma of the pancreas
FFX: FOLFIRINOX
GnP: Gemcitabine plus nab-paclitaxel
C-CAT: Center for Cancer Genomics and Advanced Therapeutics
HRR: Homologous recombination repair
NCC: OncoGuide™ NCC Oncopanel System
F1CDx: FoundationOne® CDx
F1L: FoundationOne® Liquid CDx
ECOG-PS: Eastern Cooperative Oncology Group performance status
ORR: Overall response rate
DCR: Disease control rate
TTF: Time to treatment failure
MSI: Microsatellite instability
TMB: Tumor mutation burden
MMR: Mismatch repair
CGP: Comprehensive genomic profiling
