# Supplementary material for: Genomic landscape and clinical features of rare subtypes of pancreatic cancer: analysis with the national database of Japan
Source: J Gastroenterol. 2023 Apr 7;58(6):575–85. doi: 10.1007/s00535-023-01986-9 (PMC10199859; doi:10.1007/s00535-023-01986-9)
Supplement: Supplementary file 1 — Supplementary file1 (PDF 269 KB) [file 535_2023_1986_MOESM1_ESM.pdf]

Supplementary Table 1. The frequency of minor genes by the subtypes of pancreatic cancer

	ACC	ASC	ACP	PDAC	*P* value
AKT2	0	7.4	0	2.3	0.104
ALK	2.3	0	0	0.2	0.15
APC	0	0	4	1.1	0.396
ATRX	0	0	0	0.04	1
BAP1	0	1.9	0	0.3	0.34
BARD1	0	0	0	0.04	1
BRAF	2.3	3.7	4	1.5	0.17
BRAF (fusion)	13.6	0	0	0.2	<0.0001 *
BRIP1	0	0	0	0.3	1
CCND1	0	1.9	8	1.6	0.107
CCNE1	0	3.7	0	1.9	0.646
CTNNB1	13.6	0	4	0.8	<0.0001 *
CDK6	0	3.7	0	1.7	0.594
CDK12	0	0	0	0.1	1
CHEK1	0	0	0	0	-
CHEK2	0	0	0	0.9	1
EGFR	2.3	0	0	0.4	0.242
ERBB2	4.6	1.9	0	2.4	0.586
FANCA	0	0	0	0.3	1
FANCC	0	0	0	0.08	1
FANCL	0	1.9	0	0.2	0.209
FBXW7	0	1.9	0	0.7	0.609
FGF19	0	1.9	8	1.3	0.075
FGFR1	2.3	5.6	4	1.4	0.045
FGFR2	0	0	0	0.2	1
FGFR3	0	1.9	0	0.4	0.403
FGFR4	0	0	0	0.08	1
GNAS	2.3	1.9	8	2.6	0.286
IDH1	0	0	0	0.1	1
KMT2D	2.3	3.7	12	1.8	0.014 †
MAP2K4	2.3	0	0	1.3	0.664
MDM2	0	0	4	0.8	0.343
MEN1	2.3	0	0	0.08	0.076
MET	0	0	0	0.6	1
MRE11	0	0	0	0.04	1
NBN	0	1.9	0	0	0.046
NF1	4.6	1.9	8	0.9	0.0048
NF2	2.3	0	0	0.3	0.197
NTRK1	2.3	0	0	0.2	0.173
NTRK2	0	0	0	0.08	1
NTRK3	0	0	0	0.1	1
PBRM1	4.6	1.9	0	1.2	0.158
PIK3CA	4.6	3.7	0	2	0.297
PPP2R1A	0	0	0	0.2	1
PTEN	2.3	7.4	4	0.9	0.002§
RAD51B	0	0	0	0.08	1
RAD51C	0	0	0	0.2	1
RAD51D	0	0	0	0.3	1
RAF1	2.3	0	0	0	0.026
RB1	2.3	1.9	4	1.5	0.275
ROS1	0	1.9	0	0.3	0.344
SMARCA4	0	3.7	4	1.3	0.144
TSC1	0	0	0	0.2	1
TSC2	0	0	0	0.2	1

ACC: acinar cell carcinoma, ASC: adenosquamous cell carcinoma, ACP: anaplastic carcinoma of pancreas PDAC: pancreatic ductal adenocarcinoma
Genes are listed according to alphabetical order from top to bottom.
Genes with yellow markers represent homologous recombination repair genes.
* p<0.0001 between ACC and PDAC, p<0.05 between ACC and ASC
† p=0.06 between ACP and PDAC
§ p<0.05 between ASC and PDAC

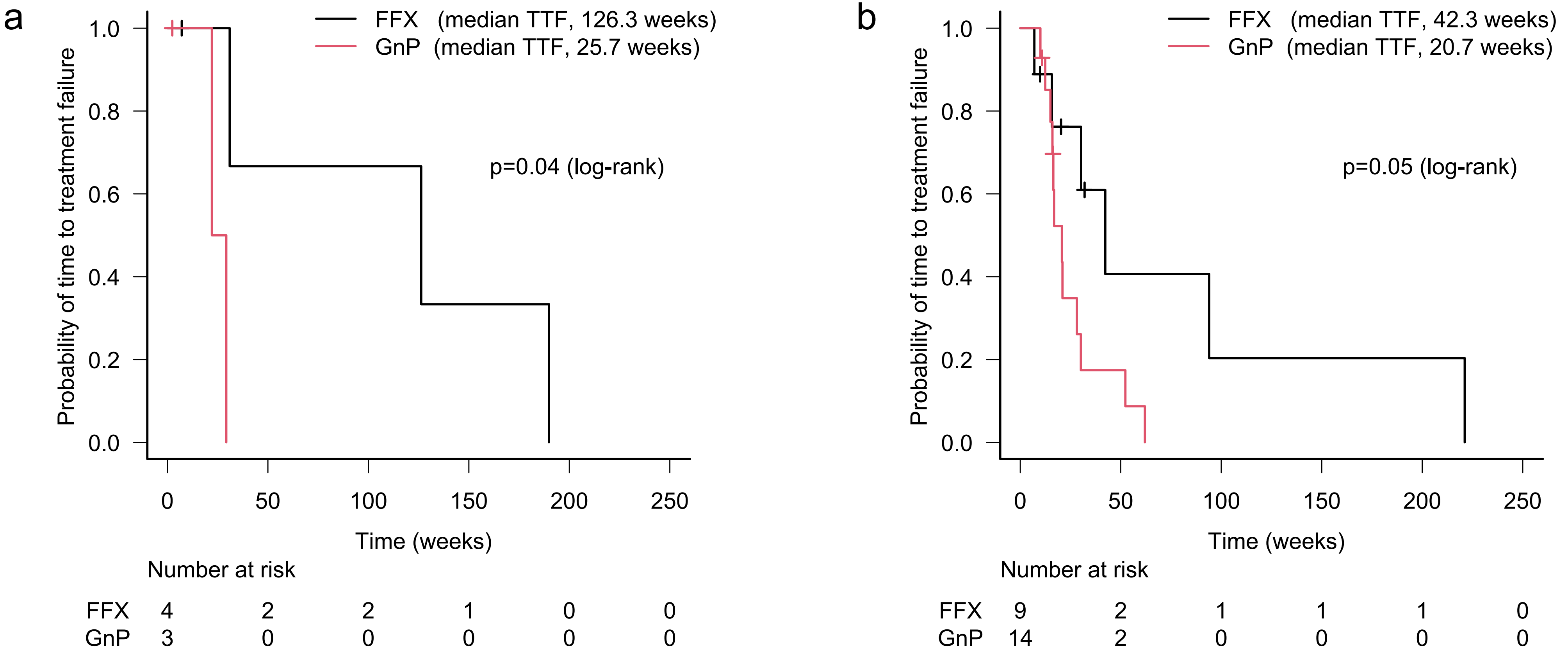

Supplementary Fig.1

Kaplan-Meier curves of time to treatment failure (TTF) according to first-line FOLFIRINOX (FFX) versus gemcitabine plus nab-paclitaxel (GnP) therapy in (a) patients with homologous recombination repair (HRR) genes and (b) patients without HRR genes.

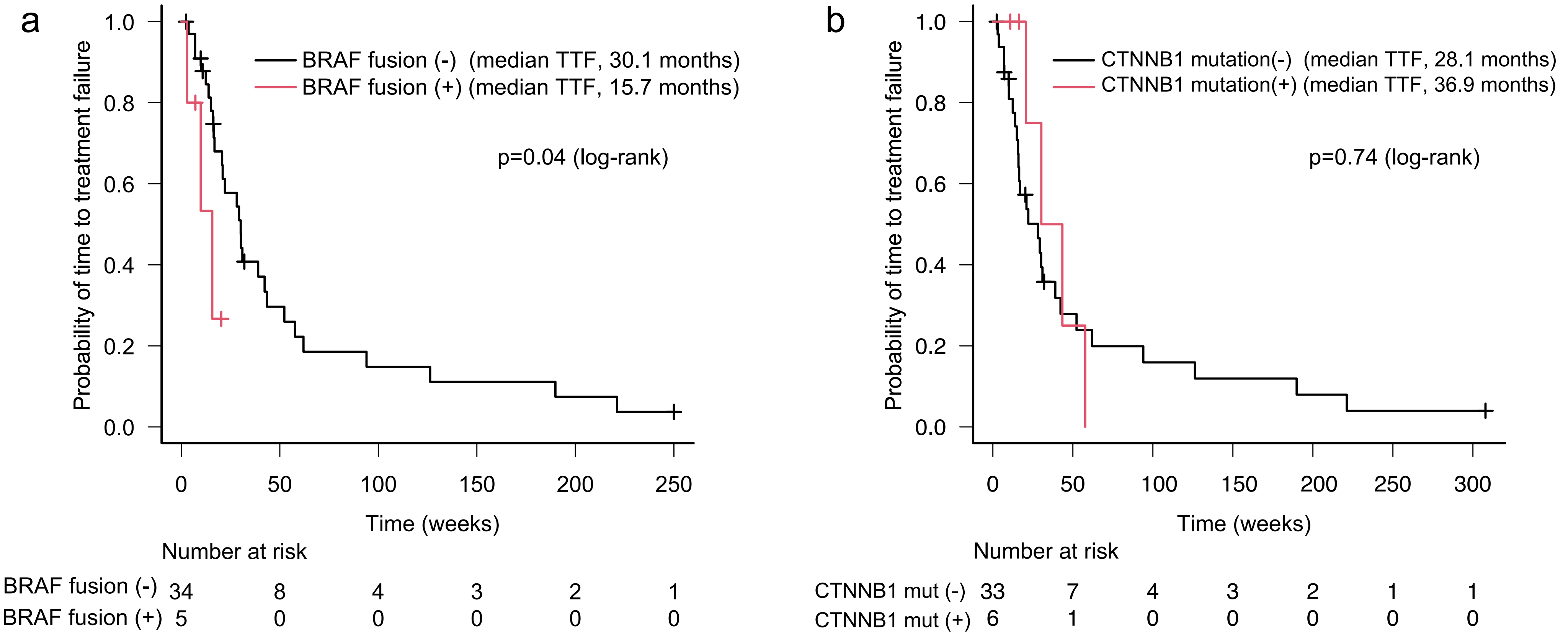

Supplementary Fig.2
Kaplan-Meier curves of time to treatment failure (TTF) according to the presence of (a) BRAF fusion and (b) CTNNB1 mutation.
